# Unusual Type of Pulmonary Stenosis Evaluated by 2D and 3D Transesophageal Echocardiography

**Published:** 2018-07

**Authors:** Ali Hosseinsabet

**Affiliations:** *Tehran Heart Center, Tehran University of Medical Sciences, Tehran, Iran.*

**Keywords:** *Pulmonary valve stenosis*, *Echocardiography*, *Echocardiography, three-dimensional*, *Echocardiography, transesophageal*

A 19-year-old male patient was referred to our echocardiography department for a better evaluation of his pulmonary stenosis. His chief complaint was dyspnea on exertion (New York Heart Association functional class II). Physical examinations revealed the presence of pectus excavatum and systolic murmurs at the right parasternal border with II/VI severity. Transthoracic echocardiography was not significantly informative due to the chest deformity; accordingly, 2D and 3D transesophageal echocardiography was done, which demonstrated thickening of the pulmonary valve cusps and restriction of their motion. Additionally, the tip of the posterolateral pulmonary cusp was attached to the sinotubular junction, resulting in the malcoaptation of the pulmonary valve cusp, moderately severe pulmonary regurgitation, and stenosis (peak pressure gradient=62 mmHg) (Figures 1 & 2). In some cases, evaluation of the pulmonary valve via transesophageal echocardiography is more informative and comprehensive than that via transthoracic echocardiography.

**Figure 1:**
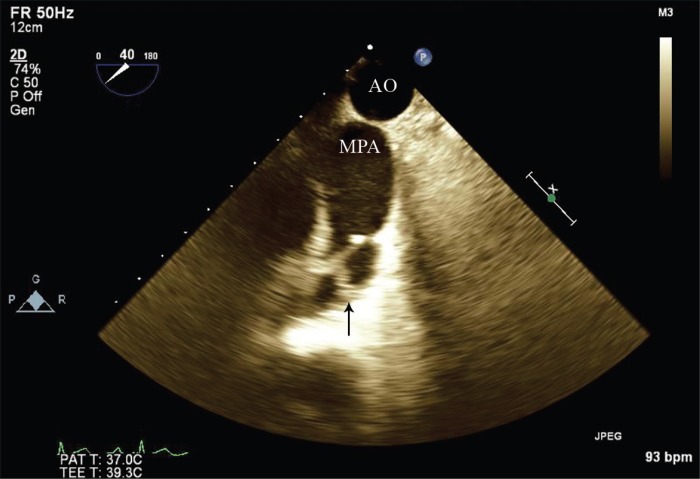
Two-dimensional transesophageal echocardiography at 40° upper esophageal view, showing that the tip of 1 pulmonary cusp is attached to the sinotubular junction (Arrow).

AO, Aorta; MPA, Main pulmonary artery

**Figure 2:**
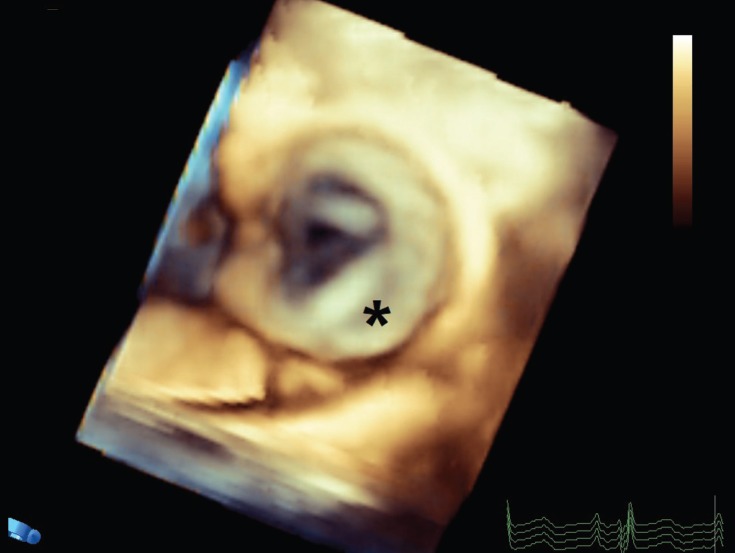
En face view of the pulmonary valve in 3D transesophageal echocardiography, showing that the tip of 1 pulmonary cusp is attached to the sinotubular junction (*).

***To watch the following videos, please refer to the relevant URLs:***

Video 1. Attachment of 1 pulmonary cusp to the sinotubular junction in 2D transesophageal echocardiography

http://jthc.tums.ac.ir/index.php/jthc/article/view/804/786

Video 2. Attachment of 1 pulmonary cusp to the sinotubular junction in 3D transesophageal echocardiography (long axis)

http://jthc.tums.ac.ir/index.php/jthc/article/view/804/787

Video 3. Attachment of 1 pulmonary cusp to the sinotubular junction in 3D transesophageal echocardiography (short axis)

http://jthc.tums.ac.ir/index.php/jthc/article/view/804/788

## Notes:

***This paper should be cited as: ***Hosseinsabet A. Unusual Type of Pulmonary Stenosis Evaluated by 2D and 3D Transesophageal Echocardiography. J Teh Univ Heart Ctr 2018;13(3):153-154.

